# PTPN22.6, a Dominant Negative Isoform of PTPN22 and Potential Biomarker of Rheumatoid Arthritis

**DOI:** 10.1371/journal.pone.0033067

**Published:** 2012-03-12

**Authors:** Hui-Hsin Chang, Tzong-Shyuan Tai, Bing Lu, Christine Iannaccone, Manuela Cernadas, Michael Weinblatt, Nancy Shadick, Shi-Chuen Miaw, I-Cheng Ho

**Affiliations:** 1 Division of Rheumatology, Immunology, and Allergy, Department of Medicine, Brigham and Women's Hospital, Boston, Massachusetts, United States of America; 2 Department of Pulmonary and Critical Care Medicine, Brigham and Women's Hospital, Boston, Massachusetts, United States of America; 3 Harvard Medical School, Boston, Massachusetts, United States of America; 4 Graduate Institute of Immunology, National Taiwan University College of Medicine, Taipei, Taiwan; University of London, St George's, United Kingdom

## Abstract

PTPN22 is a tyrosine phosphatase and functions as a damper of TCR signals. A C-to-T single nucleotide polymorphism (SNP) located at position 1858 of human PTPN22 cDNA and converting an arginine (R620) to tryptophan (W620) confers the highest risk of rheumatoid arthritis among non-HLA genetic variations that are known to be associated with this disease. The effect of the R-to-W conversion on the phosphatase activity of PTPN22 protein and the impact of the minor T allele of the C1858T SNP on the activation of T cells has remained controversial. In addition, how the overall activity of PTPN22 is regulated and how the R-to-W conversion contributes to rheumatoid arthritis is still poorly understood. Here we report the identification of an alternative splice form of human PTPN22, namely PTPN22.6. It lacks the nearly entire phosphatase domain and can function as a dominant negative isoform of the full length PTPN22. Although conversion of R620 to W620 in the context of PTPN22.1 attenuated T cell activation, expression of the tryptophan variant of PTPN22.6 reciprocally led to hyperactivation of human T cells. More importantly, the level of PTPN22.6 in peripheral blood correlates with disease activity of rheumatoid arthritis. Our data depict a model that can reconcile the conflicting observations on the functional impact of the C1858T SNP and also suggest that PTPN22.6 is a novel biomarker of rheumatoid arthritis.

## Introduction

Recent studies have identified more than 30 SNPs that are associated with a higher risk of rheumatoid arthritis (RA). One of the SNPs is located within PTPN22 [1]. This SNP (C1858T, rs2476601) converts an arginine (R620) to a tryptophan (W620) and confers the highest risk of RA among known non-HLA genetic variations [1], [2], [3], [4]. It is also associated with several other autoimmune diseases, including lupus and autoimmune diabetes [5], [6]. PTPN22 is a non-receptor tyrosine phosphatase that is expressed preferentially in hematopoietic cells [7], [8]. It contains a catalytic domain in its N-terminus, which is followed by an inhibitory domain [9], and four proline rich domains in its C-terminus. Its substrates include several cytoplasmic signaling molecules, such as Lck, Zap70, Grb2, Csk, and Vav [10], [11], [12], [13]. Previous studies in human and mouse have established that PTPN22 is a negative regulator of the TCR signals in lymphocytes [1], [2], [14].

Several studies have indicated that the C1858T SNP creates a biochemical gain-of-function variant [1], [2], [15]. W620PTPN22 attenuates TCR-induced NFAT activity more potently than R620PTPN22 when expressed in Jurkat T cells [2]. One study however demonstrates that co-expression of W620PTPN22 and one of its substrates Csk unmasks the 1858T allele as a hypomorph [16]. A recent study further suggests that the R-to-W conversion strengthens the interaction between PTPN22 and calpain 1 protease and leads to enhanced degradation of PTPN22, thereby creating a hypomorphic protein [17]. Human T cells obtained from healthy individuals carrying the 1858T allele and expressing W620PTPN22 have been shown to be hyper-responsive or hypo-responsive to stimulation in different studies [17], [18]. Thus, the effect of the C1858T SNP on the activity of PTPN22 and on the activation of lymphocytes has remained controversial. It is also unclear how the 1858T allele increases the risk of RA. Furthermore, how the overall activity of PTPN22 is regulated is largely unknown.

Here we report the identification of a novel alternative splice form of PTPN22, namely PTP22.6. We further characterize the expression and function of PTPN22.6, and explore its clinical applications in RA.

## Methods

### Preparation of human PBMC and Th cells

Peripheral blood mononuclear cells (PBMC) of normal blood donors were isolated from buffy coats purchased from Research Blood Components, LLC (Boston, MA) through an IRB (Partners Human Research Committee)-approved protocol. Th cells were enriched from PBMC with CD4 Microbeads (120-000-440, Miltenyi Biotec, Auburn, CA).

### Cell culture and medium

Human peripheral blood Th cells described in the previous section and Jurkat cells (TIB-152™, ATCC) were cultivated in RPMI-1640. In some experiments, Th cells and Jurkat cells were stimulated with 2.5 µg/ml of plate bound anti-CD3 (Hit3a, Cat. #300314, BioLegend, San Diego, CA) and 2 µg/ml of soluble anti-CD28 (CD28.2, Cat. #302914, BioLegend) in the presence of IL-2 (50 unit/ml) for indicated amount of time. Human colonic adenocarcinoma cell line HT-29 cells (HTB38, ATCC) and human embryonic kidney cells 293T (CRL-11268™, ATCC) were cultivated in McCoy's 5A medium (Gibco, Grand Island, NY) and DMEM, respectively.

### Plasmid, transfection and luciferase assay

cDNA encoding PTPN22.1 was amplified directly from Jurkat cells with primers 5′-CGGGATCCTTGCTCTGCAGCATGGACCAAAGA-3′ and 5′-GACGTCGACGCGTTTAAATATTCCAAGTTGGTGGT-3′. cDNA clone AK310570 (PTPN22.6) was obtained from NITE Biological Resource Center (Chiba, Japan). The 3′ end of Lyp2 cDNA came from the 3′ end of a cDNA fragment amplified from Jurkat cells with primers 5′-CGGGATCCTTGCTCTGCAGCATGGACCAAAGA-3′ and 5′-GACGTCGACGCGTCTAAAGCCAAGAGAAATTTTTACC-3′, and pieced together with the 5′ end of PTPN22.1 cDNA to create the full-length Lyp2. All cDNA fragments were cloned into an N-terminal FLAG-tag expression vector pCMV-Tag 2B (Stratagene, La Jolla, CA). Transfection of 293T cells was performed with Effectene Transfection Reagent (Cat. #301427, Qiagen, Valencia, CA). Transfection of Jurkat cells was performed with electroporation with Gene Pulser II (Bio-Rad, Hercules, CA) set at 374 V/1050 µF. Luciferase activity was determined with Dual-Luciferase® Reporter Assay System (Promega, Madison, WI). Firefly lucifease activity was then normalized against Renilla luciferase activity. 3xNFAT-Luc and pTK-Renilla lucifease vector were described previously [19]. Amaxa nucleofection (Amaxa Biosystems, Gaithersburg, MD) was performed according to the manufacturer's instruction. Briefly, 5 millions human CD4+ T cells were suspended in 100 µl of Human T cell Nucleofector solution (VPA-1002) and transfected with 5 µg of plasmid DNA.

### Antibodies, Western blotting, and immunoprecipitation

The following antibodies were used: human Lyp antibody AF3428 (R&D Systems, Minneapolis, MN); Hsp90 α/β antibody H-114 (sc-7947, Santa Cruz Biotechnology, Inc. Santa Cruz, CA); FLAG antibody F3165 (Sigma-Aldrich, St. Louis, MO). PTPN22.6 antibody was generated by immunizing rabbits with a modified peptide (KC-IWEYSVLIIPENFS) corresponding to the junctional sequence between exon 4 and 10. Immunoprecipitation of PTPN22.6 was performed by incubating 500 µg of whole Jurkat cell lysate with 2 µg of anti-Lyp and control goat IgG (sc-2028, Santa Cruz Biotechnology).

### Real-Time PCR

RNA isolation, reverse transcription, and real-time PCR were performed as described [20]. The following primer pairs were used: “total” 5′-GCAGAAGTTCCTGGATGAG-3′ and 5′-TCAGCCACAGTTGTAGGATAG-3′; PTPN22.1 5′- TGCCCACCAAACAAGCC-3′ and 5′-TGGTGGTGGATTCCTTGG-3′; PTPN22.6 5′-TTTGCCCTATGATTATAGCCG-3′ and 5′-GTTCTCAGGAATTATAAGGACACT-3′; β-actin 5′-GTGACAGCAGTCGGTTGGAG-3′ and 5′-AGGACTGGGCCATTCTCCTT-3′. The efficiency of the primers is 97.5% for “total”, 100.3% for PTPN22.1, 104.1% for PTPN22.6, and 98.2% for β-actin. The efficiency was calculated with MxPro™ QPCR software (Stratagene). The recommended efficiency is between 90% and 110%. All real-time PCR reactions were done in duplicate using Brilliant II SYBR Green QPCR Master Mix (Cat. #600828, Stratagene) on a Stratagene Mx3000P qPCR machine.

### siRNA transfection

One million Jurkat cells were resuspended with 400 µl Opti-mem I containing 400 pmole of siRNA and subjected to electroporation with Gene Pulser II (Bio-Rad) set at 250 V/400 µF. Human PTPN22 ON-TARGETplus SMARTpool siRNA (L-008066-00-0005) and ON-TARGETplus Non-targeting siRNA (D-001810-01-05) were purchased from Dharmacon/Thermo Scientific (Lafayette, CO).

### ELISA

IL-2 production was quantified by sandwich ELISA using anti-human IL-2 (Cat. #555051) and biotinylated anti-human IL-2 (Cat. #555040) purchased from BD Pharmingen (San Diego, CA).

### Rheumatoid arthritis samples and statistical analysis

The Brigham and Women's Hospital Rheumatoid Arthritis Sequential Study (BRASS) Registry has been described previously [21]. All participants were recruited through an IRB (Partners Human Research Committee)-approved written informed consent. The diagnoses of RA were verified according to 1987 ACR criteria. Whole peripheral blood was collected in PAXgene tubes (Qiagen). Total RNA was then prepared from the PAXgene tubes according to the manufacturer's instructions. Disease activity was determined with DAS28-CRP3 (Disease Activity Score based on 28 joint counts with 3 variables including C-reactive protein) and was calculated based on the equation DAS28-CRP3 = [0.56×√(tender joint count)+0.28×√(swollen joint count)+0.36 log_nat_(CRP+1)]×1.10+1.15. Correlation between the level of PTPN22 isoforms and DAS28-CRP3 was determined with Linear Regression Models considering gender, status of RF, and status of anti-CCP as variables.

## Results

### PTPN22.6 is a novel alternative splice form of PTPN22

**Figure 1:**
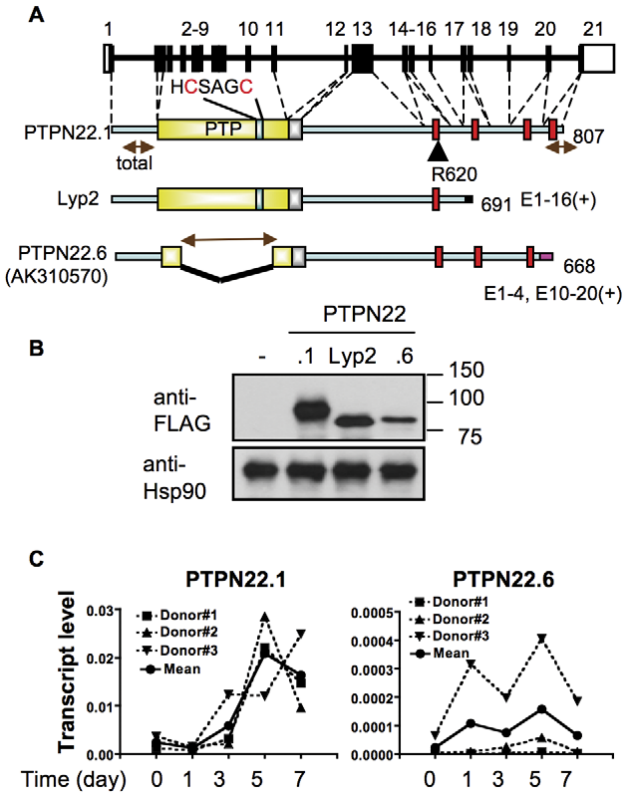
Alternatively spliced forms of human PTPN22. **A.** Schematic diagrams of the human *ptptn22* gene and putative protein products of each isoform. Exons are numbered and white boxes represent un-translated exons. The yellow box represents protein tyrosine phosphatase (PTP) domain. The key catalytic region (HCSAGC) and R620 are marked. The silver box after the PTP domain is the inhibitory domain. The red boxes represent proline rich regions. The brown two-headed arrows indicate the amplification products of real-time PCR for each isoform. The exons constituting each isoform are indicated. **B.** 293T cells were transfected with an expression vector expressing indicated FLAG-PTPN22 isoforms. The protein levels of FLAG-PTPN22 isoforms and Hsp90 in the transfected cells were determined with Western blotting. The data shown is representative of three independent experiments. **C.** cDNA was prepared from human CD4+ T cells, which were stimulated in vitro with anti-CD3/anti-CD28 for indicated periods of time, and subjected to real-time PCR analysis with isoform-specific primers. The transcript levels thus obtained were normalized against those of β-actin from the same samples. The data shown are the results from three healthy donors.

A spliced variant of PTPN22, namely Lyp2, missing the C-terminal three proline rich domains has been reported [8]. Intriguingly, we have also found in NCBI Gene database a cDNA sequence (AK310570) corresponding to a novel spliced variant of human PTPN22 (Figure 1A). AK310570 lacks exons 5–9, which encode nearly the entire PTP domain. It also lacks exon 21 but includes at its C-terminus 8 novel amino acid residues encoded by the genomic sequence immediately following exon 20 (Figure S1). We named the full-length version of PTPN22 and this novel isoform PTPN22.1 and PTPN22.6, respectively. When expressed in 293T cells, FLAG-fused PTPN22.1 and Lyp2 gave rise to protein products of 92 kd and 79 kd, respectively (Figure 1B). However, the actual molecular weight of FLAG-PTPN22.6 was slightly bigger than the expected 76 kd. We were able to amplify the full length PTPN22.1 and PTPN22.6 directly from human T cells (data not shown), confirming the presence of PTPN22.6 transcript in T cells. However, we were unable to do so for full-length Lyp2 despite several attempts with various primer pairs. Thus, the existence of Lyp2 is still questionable and we decided not to further investigate Lyp2.

### Expression of PTPN22.6 in human T cells

**Figure 2:**
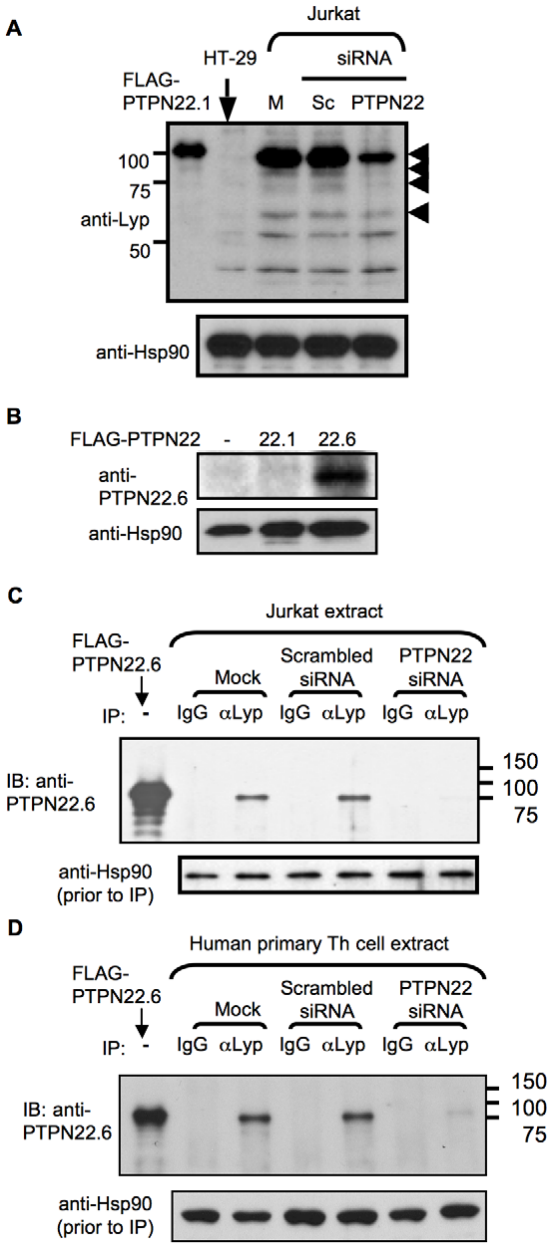
Detection of endogenous PTPN22.6 protein in human T cells. **A.** Protein extract was prepared from HT-29 or Jurkat cells, which were mock (M) transfected or transfected with PTPN22 specific siRNA or scrambled (Sc) siRNA prior to harvest. The extract was analyzed with Western blotting using Lyp antibody. The arrowheads indicate protein species that are not present in HT-29 cells and are suppressed by PTPN22 siRNA. **B.** 293T cells were transfected with the plasmid vector expressing FLAG-PTPN22.1 or FLAG-PTPN22.6. The transfected cells were then analyzed with Western blotting using PTPN22.6 and Hsp90 antibody. **C.** Protein extract prepared from Jurkat cells described in **A** was subjected to immunoprecipitation with anti-Lyp antibody or control IgG. The immunoprecipitate along with protein extract prepared from 293T cells expressing FLAG-PTPN22.6 were analyzed with Western blotting using PTPN22.6 antibody. A fraction of Jurkat cell extract was set aside prior to immunoprecipitation and was probed with Hsp90 antibody to demonstrate equal input. IP and IB stand for immunoprecipitation and immunoblot, respectively. **D.** Primary human Th cells were harvested from healthy donors and expanded in vitro with anti-CD3/atni-CD28 for 5 days. The cells were then transfected with siRNA and subjected to immunoprecipitation as Jurkat cells described in **A** and **C**. The data shown in **A**, **B**, and **C** are representative of at least two independent experiments.

To examine the expression kinetics of PTPN22.6 in human T cells, we prepared cDNA from human primary CD4+ T cells that were stimulated with anti-CD3/anti-CD28 for various periods of time. The cDNA was then subjected to real-time PCR with primers specific to PTPN22.1 and PTPN22.6 (Figure 1A). We also designed a pair of primer targeting the 5′ region shared by both PTPN22.1 and PTPN22.6 to quantify “total” PTPN22 expression. The results thus generated with the “total” and PTPN22.1 primers were very comparable, indicating that PTPN22.1 is the dominant species of PTPN22 transcripts. We henceforth used “total” and PTP22.1 primers interchangeably. We measured the level of PTPN22.1 and PTPN22.6 in T cells obtained from three healthy donors. We found that the level of PTPN22.1 transcript gradually increased and eventually peaked 5 days after stimulation (Figure 1C). The level of PTPN22.1 transcript was approximately 100 times more than that of PTPN22.6, which was only modestly induced anti-CD3 stimulation. Both PTPN22 transcript was present at a very low (10^−9^ to 10^−10^ relevant to β-actin) or undetectable level in a human colonic adenocarcinoma cell line, HT-29, which does not express PTPN22 (data not shown and Figure 2A). Although the level of PTPN22.1 or “total” transcript was very comparable among the three donors, the level of PTPN22.6 of donor #3 was approximately 60 times and 20 times of that of donor #1 and donor #2, respectively, at almost all time pointes. The efficiency of the primer pairs used in Figure 1C is very comparable (see Methods). Therefore, the influence of primer efficiency on the transcript level shown in Figure 1C is negligible.

### Detection of the protein product of PTPN22.6 in human T cells

We found that a commercially available antibody, anti-Lyp, that is specific to human PTPN22 recognized several protein species in human Jurkat T cells but not in HT-29 cells (Figure 2A). The level of at least three (and possibly four) of the protein species, including a dominant band corresponding to PTPN22.1, was reduced in extract prepared from Jurkat cells transfected with PTPN22 siRNA but not scrambled siRNA (Figure 2A), suggesting that these three or four protein species are either alternatively spliced products of PTPN22 or degradation products of PTPN22.1. The PTPN22 siRNA contains four sequences. Three of the four sequences are shared by both PTPN22.1 and PTPN22.6. To confirm the presence of endogenous PTPN22.6 protein, we raised a rabbit antiserum specific to PTPN22.6. The PTPN22.6 antibody thus generated was able to recognize exogenous PTPN22.6 but not PTPN22.1 (Figure 2B). However, it failed to detect endogenous PTPN22.6 (data not shown) probably because of low protein level of endogenous PTPN22.6 and/or poor avidity of the antibody. We then subjected Jurkat cell extract to immunoprecipitation with anti-Lyp antibody. The immunoprecipitate was then probed with anti-PTPN22.6. Immunoprecipitation with anti-Lyp antibody yielded a dominant protein band of expected molecular weight of PTPN22.6 (approximately 76 kd), which migrated slightly faster than FLAG-PTPN22.6 and was recognized by anti-PTPN22.6 (Figure 2C). The level of this protein band was markedly reduced in Jurkat cells transfected with PTPN22 siRNA but not scrambled siRNA. No protein band with a molecular weight equivalent to that of PTPN22.1 (approximately 92 kd) was detected. Thus, this 76 kd protein is not a degradation product of PTPN22.1. Similar results were obtained when Jurkat cell extract was first immunoprecipitated with anti-PTPN22.6 and then probed with anti-Lyp antibody (data not shown).

To further confirm that the protein product of PTPN22.6 is present in primary human Th cells, we harvested Th cells from peripheral blood of healthy donors and transfected the Th cells with PTPN22 siRNA, scrambled siRNA, or no siRNA. Cell extract of the transfected cells was then subjected to immunoprecipitation with anti-Lyp antibody. Again, we were able to precipitate a dominant protein band that was recognized by PTPN22.6 antibody (Figure 2D). The level of this protein band was reduced by PTPN22 siRNA but not scrambled siRNA. These data collectively confirm the presence of protein product of PTPN22.6 in human T cells.

### PTPN22.6 can function as a dominant negative variant of PTPN22.1

**Figure 3:**
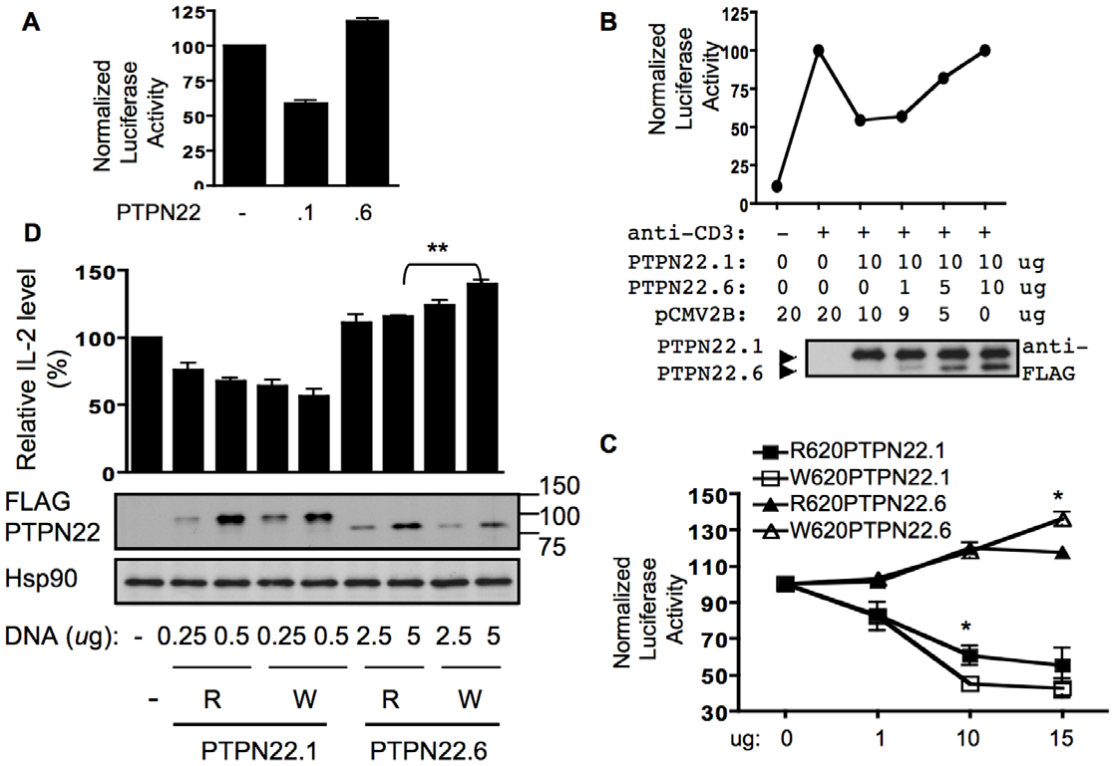
PTPN22.6 is a dominant negative variant of PTPN22.1. **A.** Jurkat cells were transfected with a NFAT-luc reporter, 10 µg of pCMV2B expressing indicated FLAG-PTPN22 isoforms, and a TK-Renilla reporter. The transfected cells were then stimulated with anti-CD3 overnight and the luciferase activity was analyzed. Normalized firefly lucifease activity was calculated as described in Methods and was shown. The data shown are cumulative results of at least three independent experiments. **B.** Jurkat cells were transfected with the NFAT-luc, TK-Renilla, 10 µg of pCMV2B-FLAG-PTPN22.1, and increasing amount of pCMV2B-FLAG-PTPN22.6. Empty pCMV2B vector was added to equalize the total amount of expression vector in each sample. Normalized luciferase activity was shown. The data shown in the top panel are cumulative results of at least three independent experiments. The standard error bars are too narrow to see. pCMV2B-FLAG-PTPN22.1 and pCMV2B-FLAG-PTPN22.6 at the same ratio was used to transfect 293T cells. The protein level of FLAG-PTPN22.1 and FLAG-PTPN22.6 in transfected 293T cells were quantified with Western blotting using FLAG antibody. One representative Western blot is shown. **C.** Jurkat cells were transfected with NFAT-luc, TK-Renilla, and increasing amount of the pCMV2B vector expressing indicated FLAG-PTPN22 protein. Transfected cells were stimulated and normalized luciferase activity was shown. The data shown are cumulative results of at least three independent experiments. *p<0.05. In all luciferase experiments shown in **A, B,** and **C**, the normalized luciferase activity obtained from cells transfected with empty pCMV2B and stimulated with anti-CD3 was arbitrarily set as 100. **D.** Primary human CD4+ T cells were transfected with indicated amount (in µg) of pCMV2B vector expressing indicated FLAG-PTPN22 variants. In some transfections, empty pCMV2B vector was added to bring up the total amount of expression vector to 5 µg. The transfected cells were stimulated with anit-CD3 (1 µg/ml) for 72 hours. The concentration of IL-2 in supernatant was measured with ELISA. The IL-2 level measured from cells transfected with empty pCMV2B vector (300–700 pg/ml) was arbitrarily set as 100%. Cell extract of the transfected cells was also probed with anti-FLAG and anti-Hsp90 antibodies in a Western blot to show the level of various FLAG-PTPN22 proteins. The data shown are cumulative results of at least three independent experiments. *p<0.05; **p<0.005. A representative Western blot is shown in the bottom panel.

PTPN22.6 does not contain the entire PTP domain and has the potential of acting as a dominant negative variant of PTPN22.1. PTPN22.1 is known to attenuate NFAT activity [2]. Overexpression of PTPN22.1 in Jurkat cells expectedly suppressed NFAT-dependent luciferase activity by approximately 50%. In contrast, PTPN22.6 modestly increased NFAT activity in a dose dependent manner (Figure 3A and 3C). To examine whether PTPN22.6 can act as a dominant negative isoform of PTPN22.1, we co-expressed PTPN22.1 and PTPN22.6 at various ratios. We found that PTPN22.6, at a protein level less than a half of that of PTPN22.1, completely nullified the effect of PTPN22.1 (Figure 3B), confirming the dominant negative nature of PTPN22.6.

### The functional impact of the C1858T SNP is isoform-dependent

It has been shown that W620PTPN22.1 was more potent than R620PTPN22.1 in suppressing NFAT activity. But this gain-of-function effect disappeared when both proteins were expressed at a high level [2]. We independently confirmed this observation (Figure 3C). Forced expression of R620PTPN22.1 attenuated NFAT-driven luciferase activity in a dose-dependent manner. W620PTPN22.1 was more efficient than R620PTPN22.1 in this assay at 10 µg of plasmid DNA but not at 15 µg dose. We then examined the impact of the C1858T SNP on NFAT activity in the context of PTPN22.6. R620PTPN22.6 enhanced NFAT activity in a dose-dependent manner but its effect peaked at 10 µg of plasmid DNA. W620PTPN22.6 comparably enhanced NFAT activity up to 10 µg of plasmid DNA but its effect continued to increase even at 15 µg of plasmid DNA. At this dose, W620PTPN22.6 was more effective than R620PTPN22.6 in enhancing NFAT activity.

We then expressed each of the four variants of PTPN22 in primary human CD4+ T cells via amaxa nucleofection. Expression of both variants of PTPN22.1 resulted in a dose-dependent reduction in IL-2 production. There was a trend of less IL-2 production with W620PTPN22.1 compared to R620PTPN22.1. However, the difference was subtle and did not reach statistical significance (Figure 3D). While R620PTPN22.6 had a negligible effect, W620PTPN22.6 enhanced the production of IL-2 in a dose dependent manner. It increased the level of IL-2 by 20–30% at a protein level similar to that of R620PTPN22.6. As approximately only a half of the cells were transfected, we estimated that expression of W620PTPN22.6 would lead to a 40–60% increase in cytokine production if we were able to achieve 100% transfection efficiency. These results indicate that the impact of the C1858T SNP on NFAT activity and T cell activation is isoform-dependent.

### The level of PTPN22.6 positively correlates with disease activity of RA

**Figure 4:**
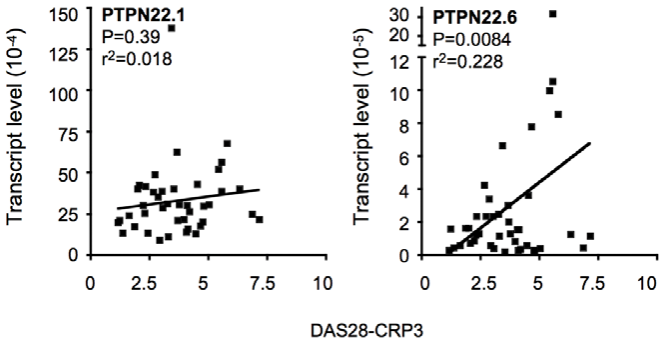
Expression of PTPN22 isoforms in RA patients. cDNA was prepared from whole blood of 41 RA patients described in Table 1. The cDNA was subjected to real-time PCR using PTPN22 isoform-specific primers. The transcript level thus obtained was normalized against that of β-actin obtained from the same sample and plotted against DAS28-CRP3. Linear regression model was used to evaluate the relationship between the transcript level and DAS28-CRP3. The *p* value for slope estimate and R^2^ of each model were shown.

Our data suggest that the overall PTPN22 activity is dependent on a functional balance between PTPN22.1 and PTPN22.6. An increase in the level of PTPN22.6 can potentially disrupt this functional balance and lead to hyperactivation of T cells, thereby contributing to the pathogenesis of RA. To test this hypothesis, we opted to examine the transcript level of PTPN22 isoforms in whole peripheral blood of patients from the Brigham and Women's Hospital Rheumatoid Arthritis Sequential Study (BRASS) cohort. We found that the transcript level determined with the PTPN22 primers was very comparable between whole peripheral blood and purified un-stimulated T cells (comparing time 0 of Figure 1C and Figure 4). We then quantified the transcript levels of PTPN22 isoforms in peripheral blood samples collected from randomly selected 41 patients (one sample per patient). The average age was 54 (Table 1). All patients except two were female. Thirty-one out of the forty-one patients (75.6%) were positive for rheumatoid factor (RF) or anti-CCP. None of these patients were genotyped for the C1858T SNP. The level of PTPN22.1 was 10 to 100 times of that of PTPN22.6. There was no difference in the level of PTPN22.1 or PTPN22.6 between sero-positive and sero-negative populations. There was also no correlation between the level of PTPN22.1 (or “total” transcript) and disease activity determined with DAS28-CRP3 (Disease Activity Score based on 28 joint counts with 3 variables including C-reactive protein). However, we found a linear and positive correlation (*p* = 0.0084) between PTPN22.6 and DAS-CRP3 even after adjusting for the potential confounding factors, such as age and status of RF and anti-CCP.

**Table 1:** Characteristics of study subjects.

Total	Age	Gender	aRF+	Anti-CCP+	RF+ or anti-CCP+
41	54±13	39F, 2M	27 (65.9%)	27 (65.9%)	31 (75.6%)

RF: rheumatoid factor; CCP: citrullinated cyclic protein.

a The status of RF in one patient is unknown.

## Discussion

Our data validate the existence of PTPN22.6 and demonstrate that it is a dominant negative variant of PTPN22.1. The expression of PTPN22.6 varies significantly among healthy individuals. It will be of great interest to investigate in the future whether T cells obtained from individuals expressing a high level of PTPN22.6, such as donor#3, will be hyper-responsive to stimulation. During the preparation of this manuscript, two additional isoforms, NM012411 (isoform 2) and NM001193431 (isoform 3), were deposited in NCBI database. We have also found in the NCBI database several cDNA sequences that may encode additional PTPN22 isoforms. The function and expression of isoform 2 (PTPN22.2) and 3 (PTPN22.3) and those potential PTPN22 isoforms has yet to be examined.

Our data provide attractive explanations for two yet-to-be-answered questions regarding the role of PTPN22 in RA. The first question is whether and how the overall activity of PTPN22 is altered in RA patients who do no carry the 1858T allele. One reasonable answer is that the overall activity of PTPN22 is normal in these patients. However, an alternative and intriguing scenario is that the activity of PTPN22 is also perturbed in this group of RA patients. A recent study indicates that the association between PTPN22 and RA cannot be all accounted for by this SNP [22]. C1858 is not polymorphic in Asian and African populations but several SNPs in the promoter region of PTPN22 are associated with RA in Han Chinese [23]. These observations strongly support the alternative explanation. Our data suggest that one plausible way of perturbing the overall activity of PTPN22 is to alter the level of PTPN22.6, and possibly other isoforms. This scenario can be examined by comparing the expression of PTPN22.6 in various subsets of immune cells between RA patients and healthy individuals.

The second and more puzzling question is why there is conflicting data regarding the effect of the 1858T allele on the activation of lymphocytes. The R-to-W conversion resulted in a gain in the phosphatase activity of PTPN22 and attenuated TCR-induced NFAT activity [2], [15]. However, co-expression of W620PTPN22 and Csk unmasked the 1858T allele as a hypomorph [16]. During the preparation of this manuscript, Zhang et al reported that the R-to-W conversion led to a hypomorphic PTPN22 due to enhanced degradation by calpain 1 [17]. We however found no appreciable effect of the C1858T SNP on the level or stability of PTPN22 proteins. Zhang et al further showed that human T cells that were homozygous for the 1858T allele were hyper-responsive to stimulation. This latter finding however contradicts a previous publication showing that the 1858T allele attenuated anti-CD3-induced calcium influx in human T cells in a gene dose-dependent manner [18].

Our data show that the impact of the C1858T SNP on T cell activation is actually isoform-dependent. Although the R-to-W conversion enhances the phosphatase activity of PTPN22.1 and attenuates NFAT activity in vitro, this conversion in the context of PTPN22.6 reciprocally boosters the activation of Th cells. This effect will be even more profound in T cells expressing a higher level of PTPN22.6, such as those from donor#3. Thus one plausible explanation for the aforementioned conflicting results is that the effect of the 1858T allele on T cell activation is dependent on the ratio between PTPN22.1 and PTPN22.6. It results in hypo-responsiveness in T cells with a low PTPN22.6/PTPN22.1 ratio but hyper-responsiveness in those with a high ratio.

Despite the positive correlation between the level of PTPN22.6 and RA activity, it needs to be mentioned that we measured the level of PTPN22 isoforms in peripheral blood instead of purified cell populations. Our real-time PCR assay did not allow us to determine the contribution of each subset of blood cells, many of which express PTPN22. It is likely that the elevated level of PTPN22.6 seen in our RA patients with higher disease activity is contributed by one single or a few subsets of blood cells. In this scenario, the level of PTPN22.6 in those subsets of blood cells will be a more sensitive indicator of disease activity than that in peripheral blood. It is also unclear whether the elevated level of PTPN22.6 is a cause or an epiphenomenon of higher RA activity. Prospective studies longitudinally monitoring the level of PTPN22.6 in RA patients will be needed to address this important question.

## Supporting Information

Figure S1 **Comparison of the peptide sequence between PTPN22.1 and PTPN22.6 (AK310570).**
(TIF)
