# Supplementary figures and images for: PTPN22.6, a Dominant Negative Isoform of PTPN22 and Potential Biomarker of Rheumatoid Arthritis
Source: PLoS One. 2012 Mar 12;7(3):e33067. doi: 10.1371/journal.pone.0033067 (PMC3299735; doi:10.1371/journal.pone.0033067)

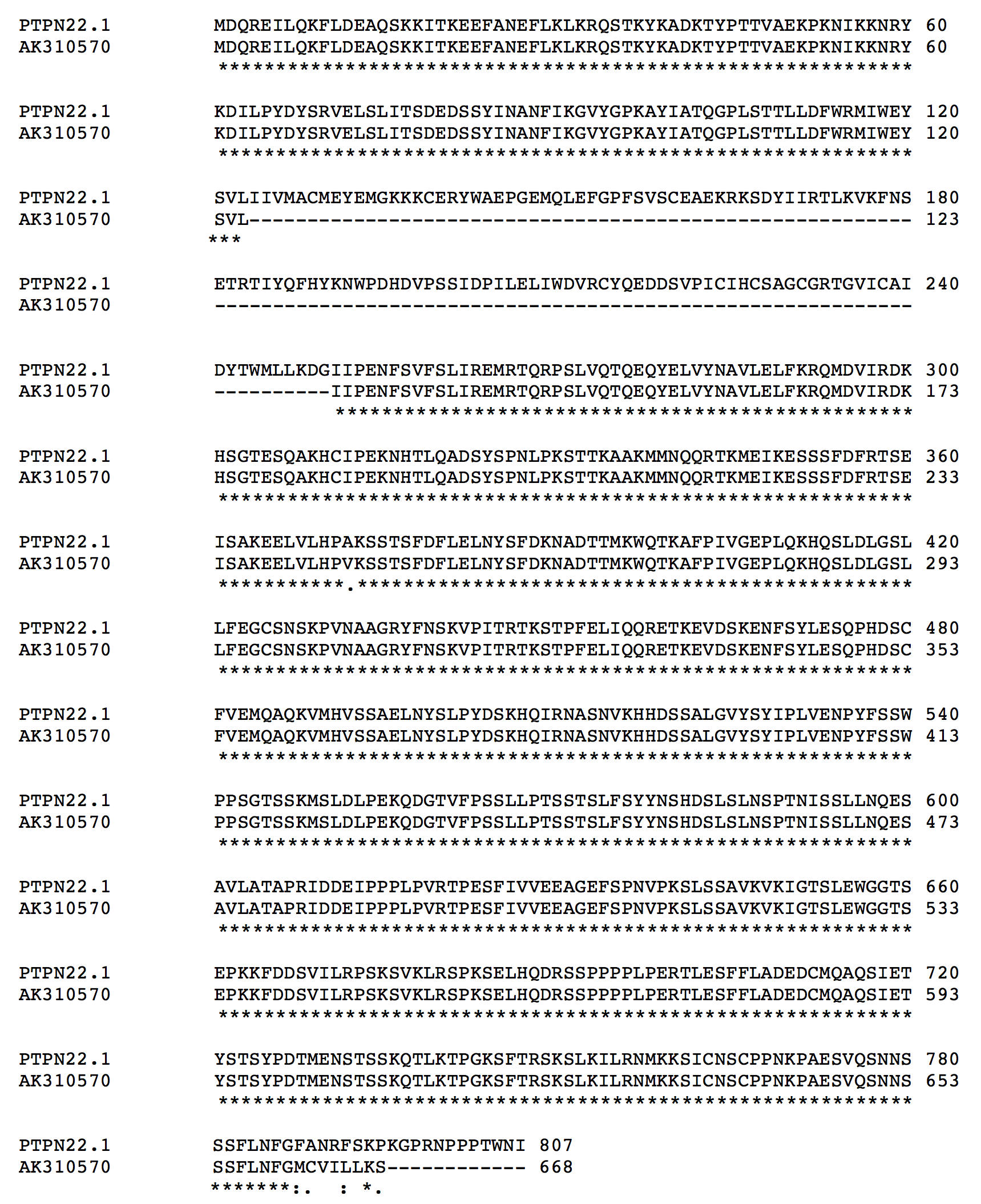

Supplement: Figure S1 — Comparison of the peptide sequence between PTPN22.1 and PTPN22.6 (AK310570). (TIF) [file pone.0033067.s001.tif]
